# Eosinophils in anti-neutrophil cytoplasmic antibody associated vasculitis

**DOI:** 10.1186/s41927-019-0059-6

**Published:** 2019-03-08

**Authors:** Thomas Hellmark, Sophie Ohlsson, Åsa Pettersson, Markus Hansson, Åsa C. M. Johansson

**Affiliations:** 1 Department of Clinical Sciences, Nephrology, Lund University, Skåne University Hospital, BMC-B13, SE-221 84 Lund, Sweden; 2 Department of Hematology, Skane University Hospital and Wallenberg Center for Molecular Medicine, Lund University, Lund, Sweden; 3 Department of Clinical Immunology and Transfusion Medicine, Lund University and Regional Laboratories Region Skane, Lund, Sweden

**Keywords:** ANCA, Systemic vasculitis, Eosinophils, Reactive oxygen species, Granulomatosis with polyangiitis, Microscopic polyangiitis, Extracellular traps

## Abstract

**Background:**

Anti-neutrophil cytoplasmic antibodies associated vasculitides (AAV) are characterized by autoimmune small vessel inflammation. Eosinophils are multifunctional cells with both pro-inflammatory and immunoregulatory properties. Tissue activated eosinophils secrete cyto- and chemokines and form extracellular traps (EETs), they release free granules and produce reactive oxygen species. The role of eosinophils is well established in eosinophilic granulomatosis with polyangiitis (EGPA) but very little is known about their role in granulomatosis with polyangiitis (GPA) and microscopic polyangiitis (MPA).

**Methods:**

The expression of surface markers CD11c, CD11b, CD16, CD35, CD62L, CD64, CD88, Siglec-8 and CD193 and reactive oxygen species production by peripheral blood eosinophils were studied using flow cytometry. Fluorescence microscopy was used to visualize the release of eosinophil extracellular DNA traps (EETs). 98 GPA and MPA patients and 121 healthy controls were included in the study.

**Results:**

Both GPA and MPA patients had decreased frequency of eosinophils in peripheral blood compared with healthy controls (*p* < 0.0001), which could not solely be explained by corticosteroid treatment. The patient’s eosinophils showed increased surface expression of the Fc receptors CD16 (*p* < 0.0001) and CD64 (*p* = 0.0035) as well as CCR3 (CD193) (*p* = 0.0022). Decreased expression was found of the complement receptors CD35 (*p* = 0.0022), CD88 (p < 0,0001) as well as CD11c (p < 0,0001), CD11b (*p* = 0.0061) and Siglec-8 (p = 0,0015). Moreover, GPA and MPA eosinophils, showed decreased capacity to produce ROS (*p* < 0.0001). ANCA stimulation of eosinophils from GPA and MPA patients after C5a priming enhanced EETosis (p = 0,0088).

**Conclusions:**

The percentage of eosinophils were decreased in peripheral blood in GPA and MPA patients and showed altered surface marker expression and function. The enhanced EETosis after ANCA stimulation, suggests that eosinophil can contribute to the autoantibody driven inflammatory process.

**Electronic supplementary material:**

The online version of this article (10.1186/s41927-019-0059-6) contains supplementary material, which is available to authorized users.

## Background

ANCA (anti-neutrophil cytoplasmic antibody) associated vasculitides (AAV) is characterized by an autoimmune small vessel inflammation [1] and autoantibodies, ANCA. AAV can be divided into three clinical diagnoses: eosinophilic granulomatosis with polyangiitis (EGPA), granulomatosis with polyangiitis (GPA) and microscopic polyangiitis (MPA).

The antigens of the autoantibodies are proteinase 3 and myeloperoxidase, that primarily are found in primary granules in neutrophils and peroxidase positive lysosomes in monocytes. Both monocytes and neutrophils are frequently found around the inflamed vessel walls and are thought to be the main effector cells. Primed neutrophils in AAV patients can be stimulated by ANCA through binding to membrane bound PR3 or MPO and in response to this they produce reactive oxygen species (ROS), de-granulates and form neutrophil extracellular traps (NETs). However, it is not known why ANCA is formed or what primes PMNs in vivo. Since eosinophils express PR3 [2] and eosinophil peroxidase (high structural homology to MPO) on their surface, ANCA might bind and activate also this cell type.

Eosinophils have for a long time been considered as nonspecific cytotoxic cells playing a role in parasitic infections and allergy. However, during the last couple of years a more complex view has evolved, stating eosinophils as multifunctional cells with for instance immunoregulatory properties [3]. At baseline, eosinophils are present in several tissues, as bone marrow, adipose tissue and gastrointestinal tract [4]. They contain more than 30 different pre-synthesized and stored proteins in their cytoplasmic granule and express receptors for proinflammatory cytokines, chemokines, lipid mediators, complement factors and immunoglobulins [5, 6]. Eosinophils could also selectively suppress Th1 cells via a constitutive expression of indoleamine 2,3-dioxygenase, an enzyme important for tryptophan catabolism [7] and they have proven to be essential for the survival of plasma cells by supplying necessary cytokines into the plasma cell niches [8].

Extracellular DNA traps formation was first described in neutrophils but is now considered as a common mechanism for the innate immune system. A main difference between NETs and EETs (Eosinophil Extracellular DNA Traps) is the association of intact granules to DNA in EETs [9]. Moreover, viable eosinophils can form EETs by releasing mitochondrial DNA and granule derived proteins [10].

We hypothesize that eosinophils are hyper reactive in situations of chronic inflammation, e.g. AAV, and can rapidly be recruited to tissues by for example innate lymphoid cell in response to microbes or other triggers. The activated eosinophil will accentuate the inflammation by releasing cyto- and chemokines, ROS production, deposition of free granules and EET formation. Moreover, neutrophils, monocytes and cells within the adaptive immune system will be recruited and give rise to additional tissue damage and pathology.

EGPA is characterized by eosinophilia and necrotizing eosinophil inflammation but very little is known about the role of eosinophils in GPA and MPA. Hence, the aims of this study are to characterize eosinophils from GPA and MPA patients, in regard to function and activation in order to understand their role in the disease.

## Methods

### Patients and controls

Non-dialysis dependent patients with MPA and GPA were recruited to the study at their scheduled visit at the outpatient clinics of Nephrology or Rheumatology, Skåne University Hospital, Lund, Sweden. The diagnosis was determined using to the method described by Watts et al. [11]. 98 GPA and MPA patients were included in the study: 74 patients with GPA, and 24 patients with MPA. Birmingham Vasculitis Activity Score version 3 (BVAS3) [12] was used to assess disease activity. Clinical characteristics are presented in Table 1. ANCA levels and specificity was performed with ELISA at Wieslab AB, Malmö, Sweden. One hundred twenty-one controls, ages 21 to 72, were collected from healthy blood donors at the Blood center in Lund and from healthy volunteers. The Regional Ethics Board in Lund, Sweden (LU) approved the study and written informed consent was obtained from all participants.

**Table 1:** Patient characteristics and demographics

	All patients (*n* = 98)	Remission^a^ (*n* = 76)	Active disease^a^ (*n* = 22)
Age, years, median (range)	53 (19–85)	68 (24–84)	63 (19–85)
Disease duration, years, median (range)	6 (0–50)	7 (0–50)	0.5 (0–25)
GPA, %, (n)	76 (74)	74 (56)	82 (18)
MPA, %, (n)	24 (24)	26 (20)	18 (4)
PR3-ANCA, %, (n)	63 (62)	61 (46)	73 (16)
MPO-ANCA, %, (n)	30 (30)	32 (24)	27 (6)
ANCA negative^b^	5 (5)	5 (4)	0 (0)
BVAS3, median (range)	0 (0–16)	0 (0–0)	5 (1–16)
Leukocytes, 10^9^/L, median (range)^c^	6.4 (3–13.7)	6.2 (3.2–13.7)	7.9 (3–12)
p-creatinine, μmol/L, median (range)	103 (54–805)	98 (61–635)	154 (54–805)
p-CRP, mg/L, median (range)	2.9 (< 0.6–92)	2.6 (< 0.6–27)	5.7 (< 0.6–92)
Treatment			
Prednisone, %, median dose of treated	59 (6.25 mg/d)	53 (5 mg/d)	82 (17.5 mg/d)
Azathioprine, % (n)	28 (27)	32 (24)	14 (3)
Mycophenolate, % (n)	8 (8)	11 (8)	0 (0)
Rituximab, % (n)	20 (20)	21 (16)	18 (4)
Methotrexate, % (n)	14 (14)	13 (10)	18 (4)
Cyklophosphamide, % (n)	13 (13)	7 (5)	36 (8)
No current treatment, % (n)	12 (12)	14 (11)	9 (3)

*BVAS3* Birmingham Vasculitis Activity Score version 3, *GPA* granulomatosis with polyangiitis, *MPA* microscopic polyangiitis, *MPO* myeloperoxidase, *ANCA* anti-neutrophil cytoplasmic antibodies, *PR3* proteinase 3, *CRP* C-reactive protein

^a^Patients in remission have BVAS3 = 0 and patients with active disease BVAS3 ≥ 1

^b^ANCA data is missing on one patient and one was double positive

^c^Reference range 3.5–8.8 109/L. Treatment at the time of sampling except for Rituximab that is reported as given at any time during the disease

### Flow cytometry

The expression of selected surface markers on phagocytes was analyzed using flow cytometry. Briefly, heparinized peripheral blood (4-6 mL) was lysed, by adding 45 mL 0.84% ammonium chloride and incubated for 10 min. The lysed blood was centrifuged for 10 min at 250 g. The cells were washed once with PBS and after centrifugation resuspended in 100 μl PBS with 0.5% BSA. The cells were divided into two tubes and incubated for 20 min with antibody mix 1 and 2 respectively (Mix 1: CD10-PECy7, CD14-V500, CD16-APC-H7, CD88-PE, CD49d-APC, CD62L-FITC, CD11b-v450, CD11C-PerCPCy5.5 and Mix 2: CD10-PECy7, CD14-PerCPCy5.5, CD16-APC-H7, CD35-FITC, CD49D-APC, CD64-v450, CD193-v500, Siglec-8-PE. All antibodies were from BD Biosciences except CD11c and Siglec-8 that were purchased from BioLegend. The cells were then washed by adding 3 mL PBS and centrifuged for 3 min at 250 g and resuspended in 25 μL PBS and analyzed using a FACSCanto II and the DIVA software (Becton Dickinson Biosciences, New York, USA). Doublet cells were excluded by plotting FSC height against FSC area and the single cells were divided into monocytes, lymphocytes and granulocytes based on FSC and SSC plots. CD14 positive cells were excluded from the granulocytes and eosinophils were selected as CD16^−^/CD10^−^ and CD49d^+^, Siglec-8^+^ and/or CD193^+^ cells. 30,000 events in the granulocyte gate were recorded. Surface expression were measured as mean fluorescence intensity (MFI) by calculating the geographic mean for the respective peak.

### Oxidative burst

Production of reactive oxygen species (ROS) in peripheral blood eosinophils was investigated using the PhagoBurst assay, (Glycotope Biotechnology, GmBH, Germany), according to the manufacturer’s protocol, after ex vivo activation with phorbol-12-myristate-13-acetate (PMA) or opsonized *E. coli*. After the fixation of the cells, they were labelled by a Siglec-8-PE antibody (BioLegend) and analyzed by flow cytometry. At least 15.000 PMN were collected based on forward and side scatter properties. Eosinophils were defined as Siglec-8^+^ granulocytes (Additional file 1). After fixation and lysing (according to the manufacturer’s protocol), it was possible to select eosinophils also by their forward and side scatter properties. No patient with ROS deficiency was observed.

### Isolation of blood eosinophils and neutrophils and detection of extracellular DNA traps

Eosinophils and neutrophils were purified from peripheral blood collected in EDTA tubes for ETosis experiments. The cells were isolated using Histopaque 1119 (Sigma) followed by Percoll (GE Healthcare) gradient following the manufacturers protocols [13]. The eosinophils were thereafter separated from the granulocytes using MACS Eosinophil Isolation Kit (Miltenyi Biotech) according to manufacturer’s instruction. The cells that were removed during the eosinophil purification step were regarded as neutrophils. Viability at isolation was determined with Trypan blue. Cytospin preparations stained with May-Grünwald Giemsa were used to determine the cell purity. (Additional file 2A and B). The purified neutrophils and eosinophils were used to measure NET/EET production after stimulation with PBS, TNFα, C5a and PMA (see below).

100 μL of purified eosinophils or neutrophils at a concentration of 1 × 10^6^ /mL in 0.5%HSA/RPMI were seeded on Poly-L-lysin treated coverslips (Sigma). Neutrophils and eosinophils from healthy controls (*n* = 7) were stimulated with PBS, C5a (150 ng/mL), TNFα (5 ng/mL) or PMA (25 ng/mL) for 180 min at 37 °C and 5% CO_2_. The cells were fixed with 2% PFA (BD Cytofix, BD Biosciences) for 10 min at room temperature. The coverslips were carefully washed three times with washing buffer (3% BSA in PBS) before the cells were permeabilized for 5 min (PBS containing 3% goat serum, 3% cold water fish gelatin, 1% BSA, 0,05% Tween20, 0,5% TritonX100) at room temperature. The coverslips were then washed carefully three times with washing buffer. To block unspecific binding coverslips were incubated for 30 min (PBS containing 3% goat serum, 3% cold water fish gelatin, 1% BSA, and 0,05% Tween) at room temperature and then washed twice with washing buffer. The cells were incubated with an anti-nucleosome-Alexa 594 (final concentration 5μg/ml, (B6.SLE-1) a kind gift from Anders Bengtsson, Lund University, labelled using Alexa Flour 594 Antibody Labeling Kit from Molecular Probes) for 1 h, 37 °C 5% CO2, and then washed three times with washing buffer. Finally, the coverslips were removed from the wells and mounted on slides with mounting medium (Prolong Gold antifade reagent with DAPI, Molecular Probes) and stored in dark at 4 °C until analysis. The slides were analyzed using a Zeiss AX10 fluorescence microscopy. Five representative photographs were taken from each slide and the percentage of cells that had released NETs/EETs were calculated by two independent persons.

### Isolation and stimulation of blood eosinophils for EET analysis after ANCA stimulation

To investigate if ANCA can stimulate eosinophils to produce EET we purified eosinophils using the MACSXpress® Eosinophil Isolation kit (Miltenyi Biotech) according to the manufacturer’s instructions. By using this kit, the eosinophils can be isolated without the density centrifugation steps and resulted in less pre-activation of the eosinophils. Briefly, erythrocytes are aggregated and sedimented, while non-targeted cells are removed by immunomagnetic depletion. The eosinophils remain in the supernatant and are carefully collected into another tube. Viability at isolation was determined with Trypan blue. Cytospin preparations stained with May-Grünwald Giemsa were used to determine the cell purity. (Additional file 2C).

The eosinophils from five healthy controls and five GPA or MPA patients were seeded on coverslips as described above. As the number of eosinophils were limited, especially in samples taken from patients, we chose C5a over TNFα to prime the eosinophils prior to IgG stimulation. The eosinophils were primed for 15 min with PBS or C5a (150 ng/mL) at 37 °C and 5% CO_2_. After priming, the eosinophils were stimulated by addition of purified IgG from ANCA positive patients (250μg/mL) (one MPO-ANCA and one PR3-ANCA), purified IgG from a healthy control (250μg/mL), PBS (negative control) and PMA (positive control – PMA was not used in combination with C5a) and incubated for 180 min at 37 °C and 5% CO_2_. After the incubation the glass were treated as described above.

### Statistical analysis

All statistical analyses were performed on GraphPad Prism 8.0 software (GraphPad Software, San Diego, CA, USA). Correlations were determined by Spearman’s correlation test and linear regression analysis. Mann-Whitney U-test was used for two group comparisons. Kruskal-Wallis and Dunn’s multiple comparisons test for three or more groups. When comparing NET and EET from the same donor Wicoxon matched-pair signed rank test was used. All *p*-values were considered significant at *p* < 0.05.

## Results

### AAV patients have decreased numbers of eosinophils

Ninety-eight patients with GPA (*n* = 74, 76%) and MPA (*n* = 24, 24%) were included in the study and clinical and demographic data at time of sampling are shown in Table 1. Most patients were PR3-ANCA positive (*n* = 62, 63%), 30 patients were MPO-ANCA positive (30%) and five were ANCA negative. ANCA specificity from one patient was missing and one was double positive. The majority of the patients were in remission (*n* = 76). Twenty-two patients had an active disease, with a median activity score according to the BVAS3 of 5 (range 1 to 16).

**Fig. 1:**
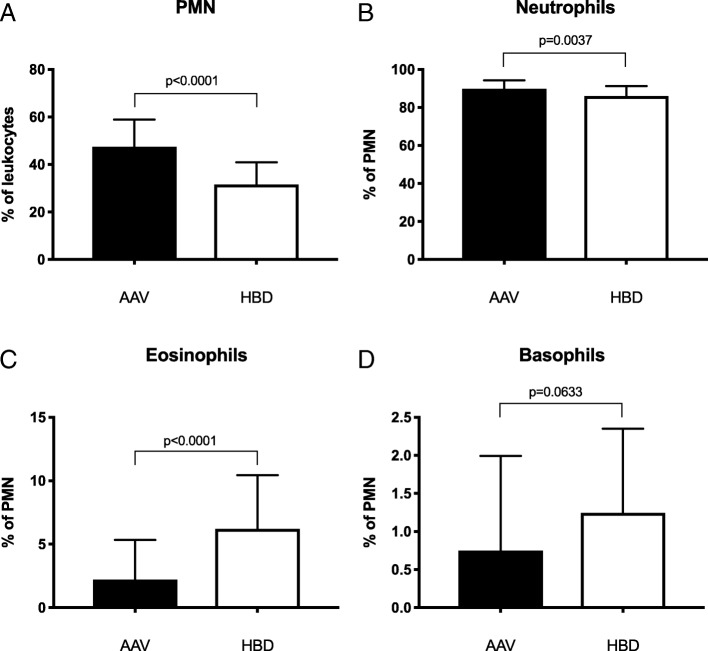
The frequencies of polymorphonuclear leukocytes (PMN) (**a**), neutrophils (**b**), eosinophils (**c**), and basophils (**d**) were investigated in healthy blood donors (HBD) and anti-neutrophil cytoplasmic antibodies associated vasculitides (AAV) patients using flow cytometry. PMNs were gated based on forward and side scatter, neutrophils as CD14^−^CD16^+^ from the PMNs, eosinophils as CD16^−^Siglec-8^+^ PMNs and the basophils were gated as side scatter low and CD193^+^ cells. Two-sided Mann-Whitney test was used to calculate the level of significance. Values are reported as median ± IQR

**Fig. 2:**
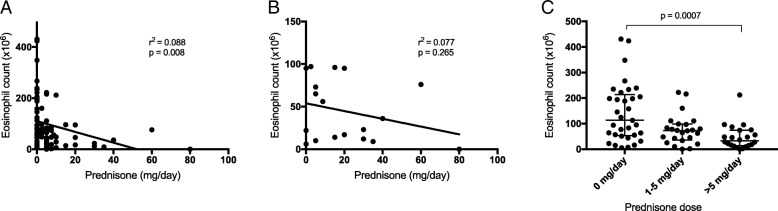
The absolute counts of eosinophils were calculated from white blood cell counts and plotted against the prednisone dose at the time of sampling. **a** GPA and MPA both active and inactive samples are plotted and in a linear regression analysis the eosinophil counts in blood show a negative correlation with the prednisone dose (p = 0.008). **b** There was no correlation when the regression analysis was performed on only patients with active disease (BVAS3 ≥ 1, p = 0.27). **c** When the all patients were divided into three groups based on their prednisone dose, we only found a significant difference between the 0 mg/day group and the one with > 5 mg/day (*p* = 0.0007). Kruskal-Wallis test and Dunn’s multiple comparisons test was used to calculate the level of significance between the three groups

The frequencies of neutrophils, eosinophils and basophils were measured in peripheral blood of patients and controls by flow cytometry. In line with our previous results [14], the patients have increased percentage of PMNs in peripheral blood (*p* < 0.0001), represented by an increased percentage of neutrophils (*p* = 0.0037, Fig. 1) In addition we observed a decreased percentage of eosinophils compared with healthy controls (*p* < 0.0001). No difference was observed in the percentage of basophils (*p* = 0.06). Since there were no white blood cell counts available for the healthy controls, we are not able to compare absolute numbers. Corticosteroid treatment has been reported to affect the number of eosinophils in peripheral blood and we saw a weak but significant correlation between corticosteroid treatment, prednisone in all of our cases, and the absolute number of eosinophils (r^2^ = 0.088, *p* = 0.008) (Fig. 2a). However, the decreased levels of eosinophils could not completely be explained by corticosteroid treatment as no correlation was found in the group with active disease (r^2^ = 0.077, *p* = 0.27) (Fig. 2b) and no significant difference of the percentage of eosinophils was observed between patients with active disease with or without corticosteroid treatment (*p* = 0.5728). When dividing all patients into 3 groups based on corticosteroid dose (0, > 0 to 5, and > 5 mg/day) the only difference found was between the group without corticosteroids and the group with a daily dose above 5 mg (Fig. 2c).

To see if disease activity or diagnosis influenced the frequencies the patients were divided into active disease (BVAS3 ≥ 1) or inactive disease (BVAS3 = 0). Patients with active disease had significantly lower frequencies of both eosinophils and basophils compared to patients with inactive disease and HBD, but no difference were found between GPA and MPA patients (Additional file 3).

### Expression of eosinophil surface markers

To characterize the status of eosinophils in peripheral blood, the expression of CD16 (FcγRIII), CD64 (FcγRI), CD35 (CR1), CD193 (CCR3), CD88 (C5aR), CD11c, CD11b, Siglec-8 and CD62L were investigated.

**Fig. 3:**
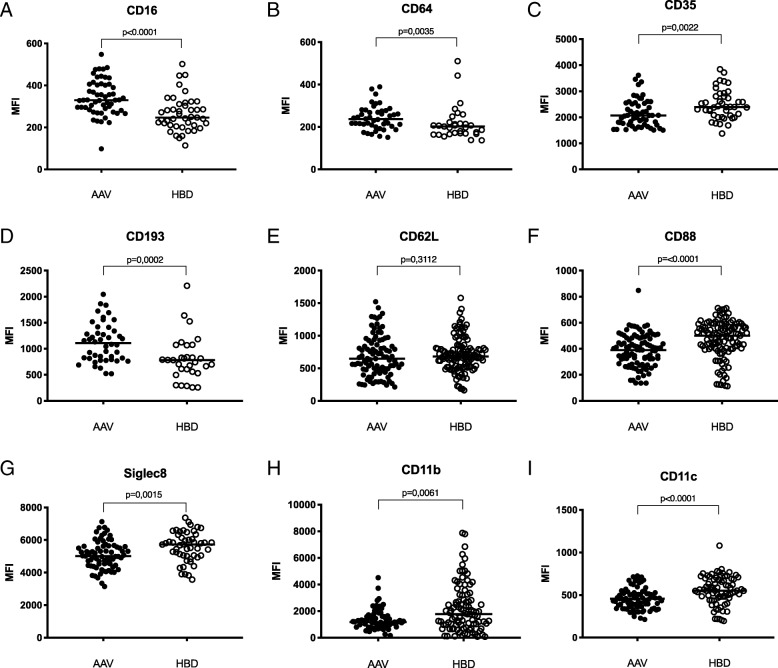
The level of surface expression on eosinophils of **a** CD16, **b** CD64, **c** CD35, **d** CD193, **e** CD62L, **f** CD88, **g** Siglec-8, **h** CD11b and **i** CD11c was measured in healthy blood donors (HBD) and anti-neutrophil cytoplasmic antibodies associated vasculitides (AAV) patients using flow cytometry and reported as geometric mean fluorescence intensity (MFI). Two-sided Mann-Whitney test was used to calculate the level of significance. The horizontal lines indicate the median values. CD16, CD64 and CD193 were found to be elevated, CD35, CD88, Siglec8, CD11b and CD11c were found to be downregulated in AAV patients

The patients have increased surface expression of the low affinity FcγRIII (CD16, *p* < 0.0001), the high affinity FcγRI (CD64, *p* = 0.0035) and the eosinophil eotaxin receptor CCR3 (CD193, *p* = 0.0002), and decreased expression of the complement receptors CD35 (*p* = 0.0022), CD88 (*p* < 0.0001) as well as CD11b (*p* = 0.0061), CD11c (*p* < 0.0001) and Siglec-8 (*p* = 0.0015) (Fig. 3). No differences were observed in the surface levels of CD62L.

**Fig. 4:**
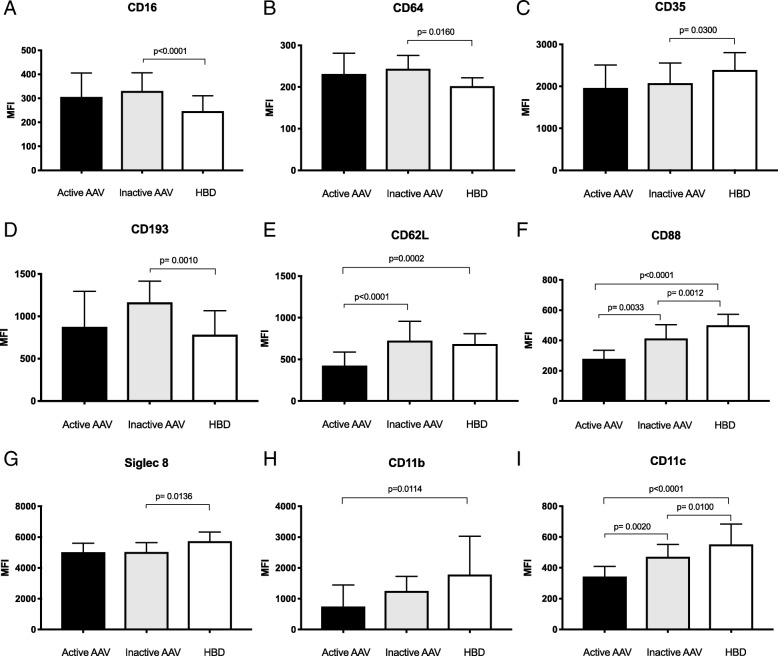
The level of surface expression on eosinophils of **a** CD16, **b** CD64, **c** CD35, **d** CD193, **e** CD62L, **f** CD88, **g** Siglec-8, **h** CD11b and **i** CD11c was measured in healthy blood donors (HBD) and compared to both active and inactive anti-neutrophil cytoplasmic antibodies associated vasculitides (AAV) patients using flow cytometry and reported as geometric mean fluorescence intensity (MFI). Kruskal-Wallis test and Dunn’s multiple comparisons test was used to calculate the level of significance between the three groups. Values are reported as median ± IQR

When the patients were divided into active (BVAS3 ≥ 1) or inactive disease (BVAS3 = 0) the patients with active disease expressed lower levels of CD88 (*p* = 0.0033), CD11c (*p* = 0.0020) and CD62L (*p* < 0.0001) compared with patients in remission (Fig. 4). No difference was seen between the GPA and MPA groups (Additional file 4).

### Decreased ROS production in eosinophils from AAV patients

**Fig. 5:**
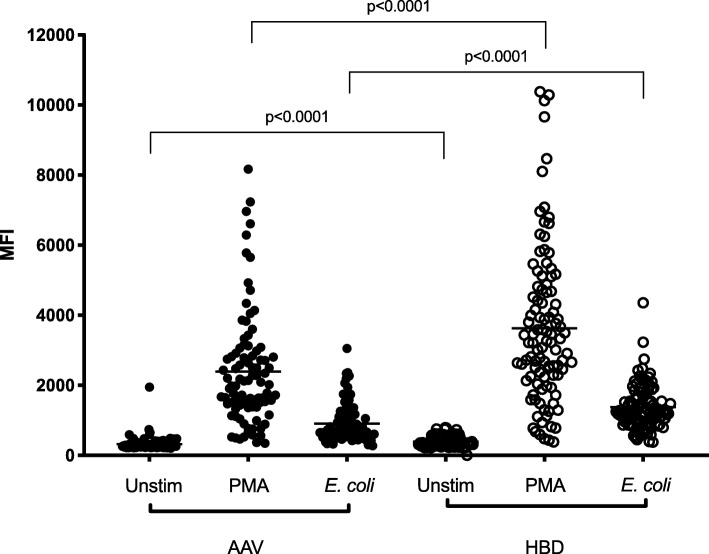
Intracellular reactive oxygen species (ROS) production in eosinophils was determined by Phagoburst kit and the produced ROS upon activation with phorbol 12-myristate 13-acetate (PMA) or opsonized *E. coli* were measured using flow cytometry and shown as geometric mean fluorescence intensity (MFI). The gating strategy and a typical pattern is shown in Additional file 1. The two-sided Mann-Whitney test was used to calculate the level of significance. Horizontal lines represent the median value of each dataset. Eosinophils from patients with anti-neutrophil cytoplasmic antibodies associated vasculitides (AAV) produced fewer ROS than eosinophils from healthy blood donors

ROS production is one of the major effector functions of phagocytes in their anti-microbial defense. Moreover, ROS play an important regulatory role of both the innate and adaptive immune system [15, 16]. Previous studies have shown that neutrophils from AAV patients have decreased intracellular ROS production. In this study we can show that this is true also for eosinophils. Peripheral whole blood from patients (*n* = 98, Table 1) and controls (*n* = 121) were stimulated with PMA (protein kinase C activator) or opsonized *E.coli* and intracellular ROS production was measured by flow cytometry using the Phagoburst kit. Eosinophils from GPA and MPA patients showed a significantly decreased ROS production both when stimulated with PMA (*p* < 0.0001) and E.coli (*p* < 0.0001, Fig. 5) compared with healthy controls.

### Eosinophils form extracellular traps more easily than neutrophils

**Fig. 6:**
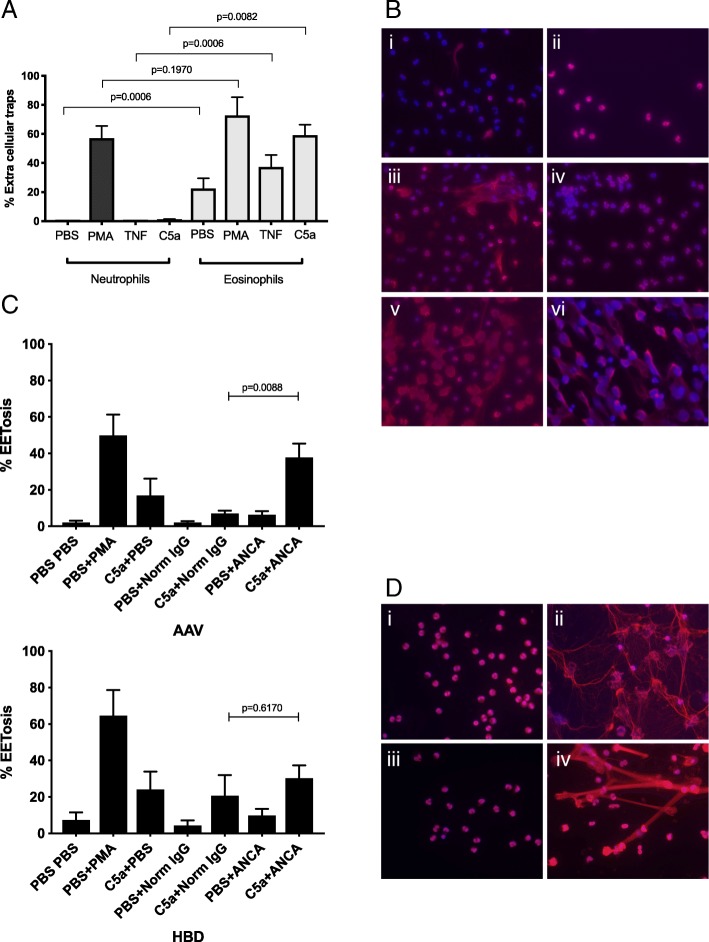
**a** Eosinophils were more prone to produce extracellular traps compared to neutrophils from the same donor (*n* = 7 healthy blood donors). Eosinophils and neutrophils were purified from peripheral blood using density centrifugation followed by MACS eosinophil isolation kit. The cells that bound to the magnetic beads are regarded as neutrophils and the ones that did not as eosinophils (Additional file 2). The cells were seeded on poly L-lysin coated cover slides, stimulated with PBS, PMA, TNF or C5a for 3 h and stained using DAPI and anti-nucleosome antibodies. The percentage of cells that had released neutrophil/eosinophil extra cellular traps (NETs/EETs) were counted under the microscope. The Wilcoxon matched pairs signed rank test was used to calculate the level of significance. **b** Shows a representative immunofluorescence experiment: DAPI is shown in blue and nucleosomes in red. Eosinophils are shown in i, iii and v and neutrophils in ii, iv and vi. In i and ii the cells were incubated with PBS, in iii and iv with C5a and in v and vi with PMA for 3 h. Cells stimulated with TNFα did not differ visually from the cells stimulated with C5a. **c** Eosinophils from anti-neutrophil cytoplasmic antibodies associated vasculitides (AAV) patients produced more EETs after stimulation with C5a and ANCA IgG compared to stimulation with C5a and IgG purified from healthy blood donors (HBD). This difference was not seen in eosinophils purified from HBD. Eosinophils were purified from peripheral blood using Eosinophils MACS Xpress kit and put on poly L-lysin coated cover slides. They were then primed for 15 min with C5a or PBS followed by the addition of purified IgG from ANCA positive patients or IgG from HBD and a 3 h incubation. They were thereafter stained using DAPI and anti-nucleosome antibodies. The percentage of cells that had undergone NET/EETosis were counted under the microscope. The two-sided Mann-Whitney test was used to calculate the level of significance. **d** Shows eosinophils from a GPA patient purified with MACSXpress kit that were incubated for 3 h at 37 °C and 5% CO_2._ In i) the cells were incubated with PBS, ii) with PMA, iii) cells that were primed with C5a for 15 min followed by purified IgG from a healthy blood donor and iv) cells that were primed with C5a for 15 min followed by purified IgG from an ANCA positive patient. DNA is visualized with DAPI (blue) and nucleosomes visualized with an anti-nucleosome antibody labelled with Alexafluor 594 (red). The EETs do generally not contain many granular proteins as the granules are released intact during EETosis (Additional file 5)

Extracellular traps released from neutrophils and eosinophils are thought to play an important role in the defense against pathogens and in inflammatory processes. Eosinophils and neutrophils were purified from peripheral blood of healthy donors (*n* = 7) and stimulated with PBS (negative control) TNFα, C5a or PMA (positive control). Eosinophils were more prone to release extracellular traps than neutrophils, when stimulated with TNFα (*p* = 0.0006) or C5a (*p* < 0.0001) but no significant difference was observed using PMA stimulation (*p* = 0.1970) (Fig. 6a and b). Eosinophils also produced more EETs in the negative control where they were incubated with PBS alone.

### ANCA stimulation enhanced extracellular trap formation in eosinophils from patients

AAV are characterized by ANCA autoantibodies that binds mainly to PR3 or MPA. Eosinophils express PR3 [2] and eosinophil peroxidase (high structural homology to myeloperoxidase) on their surface, indicating that ANCA could bind and stimulate also this cell type.

To investigate if ANCA could affect the release of EETs, eosinophils were purified using the MACSExpress eosinophil kit from five AAV patients (3 GPA and 2 MPA) and healthy controls (*n* = 5). The eosinophils were primed with either PBS or C5a followed by incubation with PBS (negative control), PMA (positive control), IgG from healthy controls or IgG from ANCA patients (one PR3-ANCA and one MPO-ANCA). Stimulation of eosinophils from the patients with C5a followed by ANCA gave a higher level of EETosis (*p* = 0,0088, Fig. 6c). This was not seen among healthy controls. EETs from these stimulations are shown in Fig. 6d. Eosinophil purification using the MACSExpress kit generated less pre-activated eosinophils from healthy blood donors as the percentage of EETs in the negative control (PBS incubation) was much lower (22% versus 5.5% in Fig. 6a and c respectively). Nonetheless, stimulation of eosinophils with C5a alone generated more EETs (61% in Fig. 6a and 24% in Fig. 6c) compared to NETs in experiments done on neutrophils (0.4% in Fig. 6a).

## Discussion

Eosinophils are multifunctional cells with both pro-inflammatory and immune-regulatory properties that have been suggested to regulate local tissue immunity, repair and remodeling [17]. Moreover, eosinophils seem to be important for B cell activation and the homing and survival of plasma cells [18]. There is some evidence that eosinophils have a protective role in autoimmunity e.g. Finlay et al. showed that activated eosinophils conferred protection against experimental allergic encephalomyelitis [19]. Here we show that the percentage of eosinophils were decreased in peripheral blood in GPA and MPA patients and showed altered surface marker expression and function. Moreover, ANCA stimulation enhanced EETosis in GPA and MPA patients a phenomenon not seen in healthy controls. The altered surface expression and low ROS production in eosinophils is true for both patients in remission and with active disease and no difference were seen between GPA and MPA patients. The results are in line with our previous findings for neutrophils in AAV patients [14].

Glucocorticosteroid treatment has been reported to affect the number of eosinophils in peripheral blood, by inducing apoptosis and inhibiting pro-survival signals by cytokines e.g. IL-5 and GM-CSF [20, 21]. In line with earlier observation we saw a weak correlation between corticosteroid treatment and the number of eosinophils in blood (r^2^ = 0.088, *p* = 0.008). However, the decreased levels of eosinophils could not completely be explained by corticosteroid treatment as no significant correlation was observed between patients with active disease with or without corticosteroid treatment (*p* = 0.27). Two patients were newly diagnosed and both of them had low levels of eosinophils already before treatment was started. Even if the pro-apoptotic effect of steroids on eosinophils is widely accepted, there are reports suggesting that it depends on the activation status of the eosinophils [22]. This could be one explanation to why we could not detect any correlation in patients with active disease. Moreover, dexamethasone has been reported to increase eosinophil differentiation and proliferation from CD34^+^ hematopoietic stem cells [23]. Why GPA and MPA patients have decreased frequencies of eosinophils in peripheral blood is not known. A possible explanation could be that activated eosinophils are recruited to sites of inflammation and play an active role in the pathogenesis of AAV. Mononuclear cells have been reported to be the most frequent cell type in interstitial infiltration of ANCA-positive renal biopsies from patients with suspected systemic vasculitis, however eosinophils and neutrophils were found in 20 and 27% of the cases respectively [24].

An important feature of eosinophils is the binding of antibodies and of complement proteins via specific receptors, events that will induce degranulation and antibody mediated cellular cytoxicity and eventually killing of invading microbes or host cells [25]. Here we showed that AAV patients have increased surface expression of the low affinity FcγRIII (CD16) as well as the high affinity FcγRI (CD64), a phenomenon described earlier in patients with allergic conditions [26]. In several conditions, including EGPA, an increased expression CD69 and CD11b and shedding of CD62L are markers of activated eosinophils [25, 27]. In this study we found less CD62L on eosinophils from patients with active disease but lower levels of CD11b. Recently, Lingblom et al. described a naturally occurring CD16^+^ eosinophil subset in peripheral blood, able to suppress T cell proliferation [28]. In line with our observation, these CD16^+^ eosinophils also showed decreased expression of some surface markers including CD88 and CD11a, whereas surface molecules associated with T cells suppression e.g. PD-L1, CD54 were up-regulated. The importance of this eosinophil subset in AAV needs further investigation.

NET depositions have been observed in kidney biopsies from AAV patients. We and others, have previously shown that ANCA could stimulate NET formation by neutrophils. Moreover, sera from AAV patients seem to degrade NETs more slowly than healthy controls [29]. Interestingly, Kraaij et al. recently showed that ANCAs did not influence NET formation by neutrophils from a healthy donor, but autologous serum induced increased formation of NETs in neutrophils from three out five studied patients [30], suggesting that it is not ANCA per se that induces enhanced NETs formation rather the combination of ANCA and neutrophils from AAV patients. In line with these results, we show that addition of ANCA increased EETs formation in eosinophils from AAV patients. How ANCA induce formation of extra cellular traps in neutrophils and eosinophils is not known. The high affinity IgG receptor FcγRI (CD64) has been shown to be induced on blood eosinophils by IFN-γ [27] and human FcγRI expressed in mice was sufficient to trigger autoimmune arthritis [31]. The high CD64 expression on eosinophils, that is further increased in AAV patients, may explain the increased EET formation in eosinophils from AAV patients compared to healthy controls when stimulated with ANCA. An increased influx and following EET formation in the inflamed tissue could partly contribute to the lower levels of circulating eosinophils. EETosis in the tissue could also mean deposition of intact eosinophilic granules (Additional file 5) in the tissue further increasing the inflammation [9]. The presence of free eosinophil granules in tissue from AAV patients still needs to be proven.

ROS are major effector molecules in inflammatory processes and tightly linked to EET formation [9, 10]. During the last decade, an increasing amount of data support an immune modulating role for monocyte and granulocyte produced ROS [15, 32–34], as ROS can affect redox sensitive pathways [35]. We have previously reported that PMN and monocytes from AAV patients had decreased capacity to phagocytose and impaired ROS production, which was associated with disease activity [14].

In this study we show that AAV eosinophils have decreased capacity to produce ROS compared with healthy controls. Even though eosinophils seem to produce sufficient amounts of ROS to form EETs, it may have an impact on immune regulation.

## Conclusions

The frequency of eosinophils was decreased in peripheral blood in AAV patients and they showed altered surface marker expression and function. They also produce less ROS when stimulated with opsonized *E.Coli* or PMA. Moreover, eosinophils produce EETs when stimulated with TNFα or C5a and addition of ANCA further increase the number of EETotic cells, suggesting that eosinophil can contribute to the autoantibody driven inflammatory process in AAV.

## Additional files

Additional file 1: Production of reactive oxygen species (ROS) in eosinophils. Cell aggregates were excluded based on forward scatter height and area properties, then granulocytes were gated based on their forward and side scatter. Eosinophils (in red) were defined as Siglec-8^+^ granulocytes. It was possible to select eosinophils also by their forward and side scatter characteristics. Intracellular production of ROS was measured as the geometric median fluorescence intensity in eosinophils (red) as a comparison typical graphs of ROS production in neutrophils are shown to the left (green). The two top histograms show unstimulated (PBS) cells and the bottom two histograms show cells activated with phorbol-12-myristate-13-acetate (PMA). (PDF 141 kb)

Additional file 2: Cytospin preparations of purified neutrophils and eosinophils stained with May-Grünwald Giemsa. In the first set of experiment neutrophils and eosinophils were isolated using Histopaque 1119 (Sigma) followed by Percoll (GE Healthcare) and thereafter the eosinophils were separated from the granulocytes using MACS Eosinophil Isolation Kit (Miltenyi Biotech). The cells that were removed from during the eosinophil purification step were regarded as neutrophils (A) and the ones that remained as eosinophils (B). In the second part of the experiment eosinophils were purified using the MACSXpress® Eosinophil Isolation kit (Miltenyi Biotech) (C). (PDF 199 kb)

Additional file 3: The percentage of eosinophils of polymorphonuclear leukocytes (PMN) and basophils of the leukocytes populations is shown when the patients are divided into active and inactive (A and B) or into GPA or MPA (C and D). Patients with active disease had lower levels of both eosinophils and basophils but no difference were seen comparing GPA and MPA. Kruskal-Wallis test and Dunn’s multiple comparisons test was used to calculate the level of significance between the three groups. Values are reported as median ± IQR. (PDF 86 kb)

Additional file 4: The level of surface expression on eosinophils of A CD16, B CD64, C CD35, D CD193, E CD62L, F CD88, G Siglec-8, H CD11b and I CD11c was measured in healthy blood donors (HBD) and compared to anti-neutrophil cytoplasmic antibodies associated vasculitides (AAV) patients, divided into GPA and MPA patients, using flow cytometry and reported as geometric mean fluorescence intensity (MFI). Kruskal-Wallis test and Dunn’s multiple comparisons test was used to calculate the level of significance between the three groups. Values are reported as median ± IQR. No difference was seen between the GPA and MPA groups. (PDF 95 kb)

Additional file 5: Light microscopy picture of two eosinophils that has formed EETs after incubation with PMA for 3 h at 37 °C and 5%CO_2_. The white arrow indicates the web formed by the DNA and the black arrows indicate the intact granules that remains around the plasma membrane remnants. (PDF 263 kb)

## Abbreviations

AAV: Anti-neutrophil cytoplasmic antibodies associated vasculitides
ANCA: Anti-neutrophil cytoplasmic antibodies
BVAS3: Birmingham Vasculitis Activity Score version 3
C5a: Complement Component 5a
C5aR: Complement Component 5a receptor
CCR3: C-C chemokine receptor type 3
CD: Cluster of differentiation
CR1: Complement receptor 1
DNA: Deoxyribonucleic acid
EDTA: Ethylenediaminetetraacetic acid
EETs: Eosinophil extracellular traps
EGPA: Eosinophilic granulomatosis with polyangiitis
ELISA: Enzyme Linked immunosorbent assay
ETs: Extra cellular traps
FCγR: Immunoglobulin gamma Fc receptor
GM-CSF: Granulocyte monocyte-colony stimulating factor
GPA: Granulomatosis with polyangiitis
HBD: Healthy blood donor
HSA: Human serum albumin
IFN: Interferon
IgG: Immunoglobulin G
IQR: Inter quartile range
MACS: Magnetic-activated cell sorting
MFI: Geographic mean fluorescence intensity
MPA: Microscopic polyangiitis
MPO: Myeloperoxidase
NADPH: Nicotinamide adenine dinucleotide phosphate
NETs: Neutrophil extra cellular traps
PBS: Phosphate buffered saline
PD-L1: Programmed death-ligand 1
PFA: Paraformaldehyde
PMA: Phorbol-12-myristate-13-acetate
PNM: Polymorphonuclear leukocytes
PR3: Proteinase 3
ROS: Reactive oxygen species
RPMI: Roswell Park Memorial Institute medium
Th1: T helper cell 1
TNF: Tumor necrosis factor
