# Supplementary material for: Eosinophils in anti-neutrophil cytoplasmic antibody associated vasculitis
Source: BMC Rheumatol. 2019 Mar 8;3:9. doi: 10.1186/s41927-019-0059-6 (PMC6408823; doi:10.1186/s41927-019-0059-6)
Supplement: Supplementary file 1 — Production of reactive oxygen species (ROS) in eosinophils. Cell aggregates were excluded based on forward scatter height and area properties, then granulocytes were gated based on their forward and side scatter. Eosinophils (in red) were defined as Siglec-8+ granulocytes. It was possible to select eosinophils also by their forward and side scatter characteristics. Intracellular production of ROS was measured as the geometric median fluorescence intensity in eosinophils (red) as a comparison typical graphs of ROS production in neutrophils are shown to the left (green). The two top histograms show unstimulated (PBS) cells and the bottom two histograms show cells activated with phorbol-12-myristate-13-acetate (PMA). (PDF 141 kb) [file 41927_2019_59_MOESM1_ESM.pdf]

Additional file 1

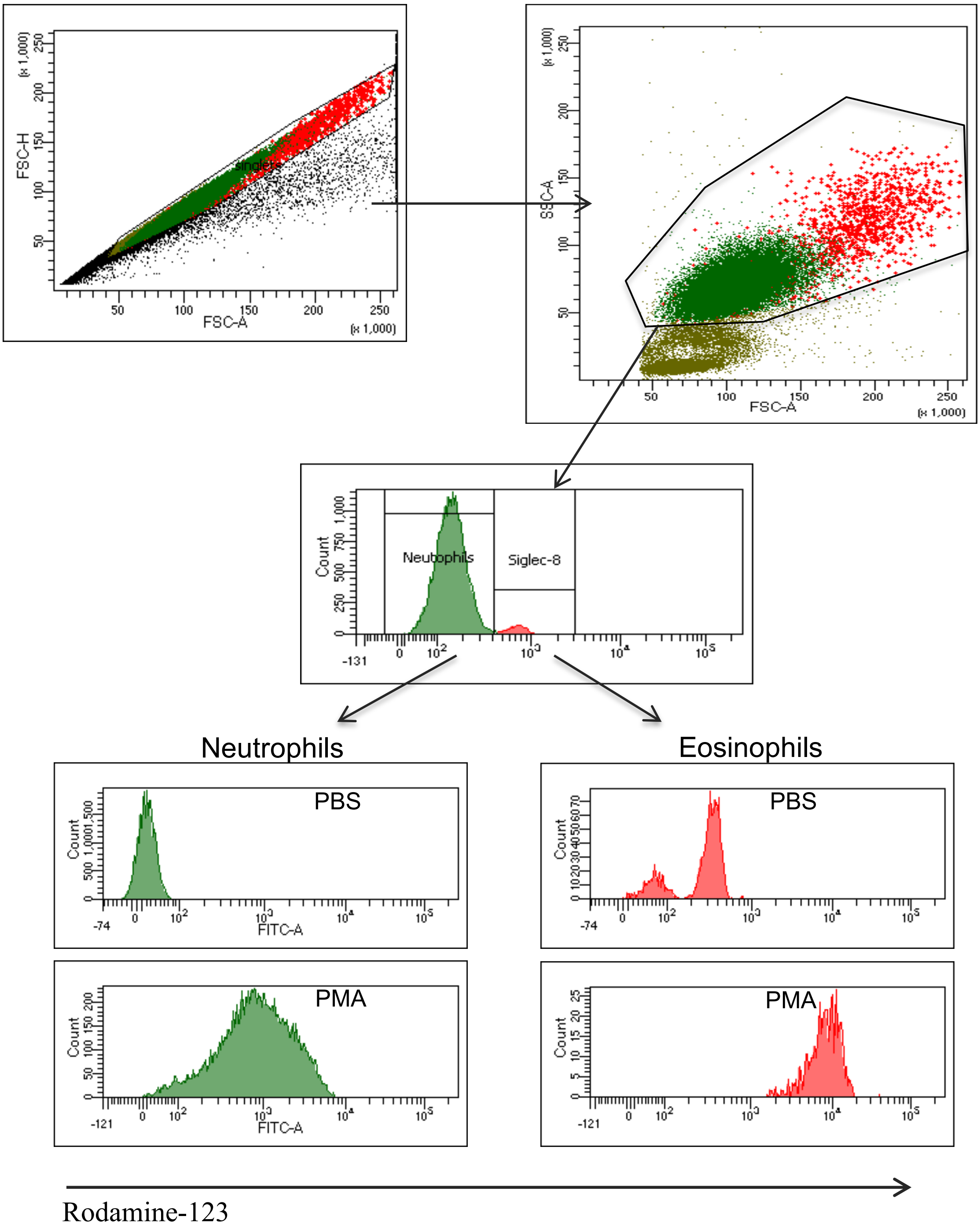

Additional file 1. Production of reactive oxygen species (ROS) in eosinophils.
Cell aggregates were excluded based on forward scatter height and area properties, then granulocytes were gated based on their forward and side scatter. Eosinophils (in red) were defined as Siglec-8^{+} granulocytes. It was possible to select eosinophils also by their forward and side scatter characteristics. Intracellular production of ROS was measured as the geometric median fluorescence intensity in eosinophils (red) as a comparison typical graphs of ROS production in neutrophils are shown to the left (green). The two top histograms show unstimulated (PBS) cells and the bottom two histograms show cells activated with phorbol-12-myristate-13-acetate (PMA).
