# Supplementary material for: Eosinophils in anti-neutrophil cytoplasmic antibody associated vasculitis
Source: BMC Rheumatol. 2019 Mar 8;3:9. doi: 10.1186/s41927-019-0059-6 (PMC6408823; doi:10.1186/s41927-019-0059-6)
Supplement: Supplementary file 2 — Cytospin preparations of purified neutrophils and eosinophils stained with May-Grünwald Giemsa. In the first set of experiment neutrophils and eosinophils were isolated using Histopaque 1119 (Sigma) followed by Percoll (GE Healthcare) and thereafter the eosinophils were separated from the granulocytes using MACS Eosinophil Isolation Kit (Miltenyi Biotech). The cells that were removed from during the eosinophil purification step were regarded as neutrophils (A) and the ones that remained as eosinophils (B). In the second part of the experiment eosinophils were purified using the MACSXpress® Eosinophil Isolation kit (Miltenyi Biotech) (C). (PDF 199 kb) [file 41927_2019_59_MOESM2_ESM.pdf]

Additional file 2

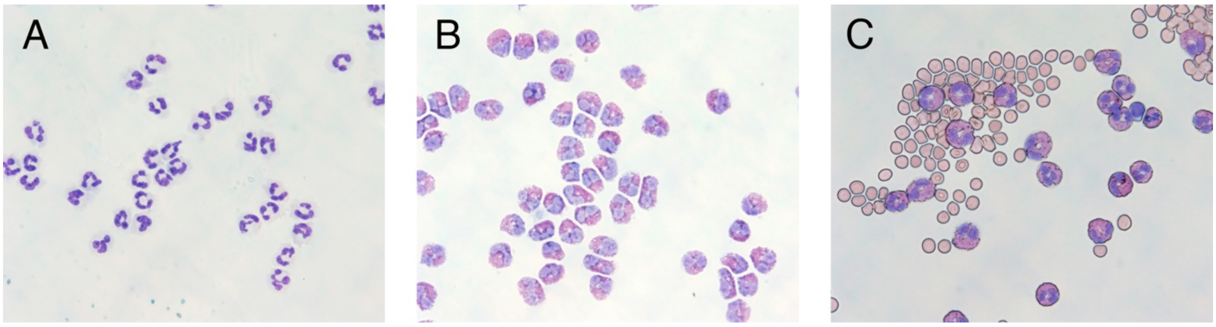

Additional file 2. Cytospin preparations of purified neutrophils and eosinophils stained with May-Grünwald Giemsa. In the first set of experiment neutrophils and eosinophils were isolated using Histopaque 1119 (Sigma) followed by Percoll (GE Healthcare) and thereafter the eosinophils were separated from the granulocytes using MACS Eosinophil Isolation Kit (Miltenyi Biotech). The cells that were removed from during the eosinophil purification step were regarded as neutrophils (A) and the ones that remained as eosinophils (B). In the second part of the experiment eosinophils were purified using the MACSXpress® Eosinophil Isolation kit (Miltenyi Biotech) (C).
