# Supplementary material for: Eosinophils in anti-neutrophil cytoplasmic antibody associated vasculitis
Source: BMC Rheumatol. 2019 Mar 8;3:9. doi: 10.1186/s41927-019-0059-6 (PMC6408823; doi:10.1186/s41927-019-0059-6)
Supplement: Supplementary file 3 — The percentage of eosinophils of polymorphonuclear leukocytes (PMN) and basophils of the leukocytes populations is shown when the patients are divided into active and inactive (A and B) or into GPA or MPA (C and D). Patients with active disease had lower levels of both eosinophils and basophils but no difference were seen comparing GPA and MPA. Kruskal-Wallis test and Dunn’s multiple comparisons test was used to calculate the level of significance between the three groups. Values are reported as median ± IQR. (PDF 86 kb) [file 41927_2019_59_MOESM3_ESM.pdf]

Additional file 3

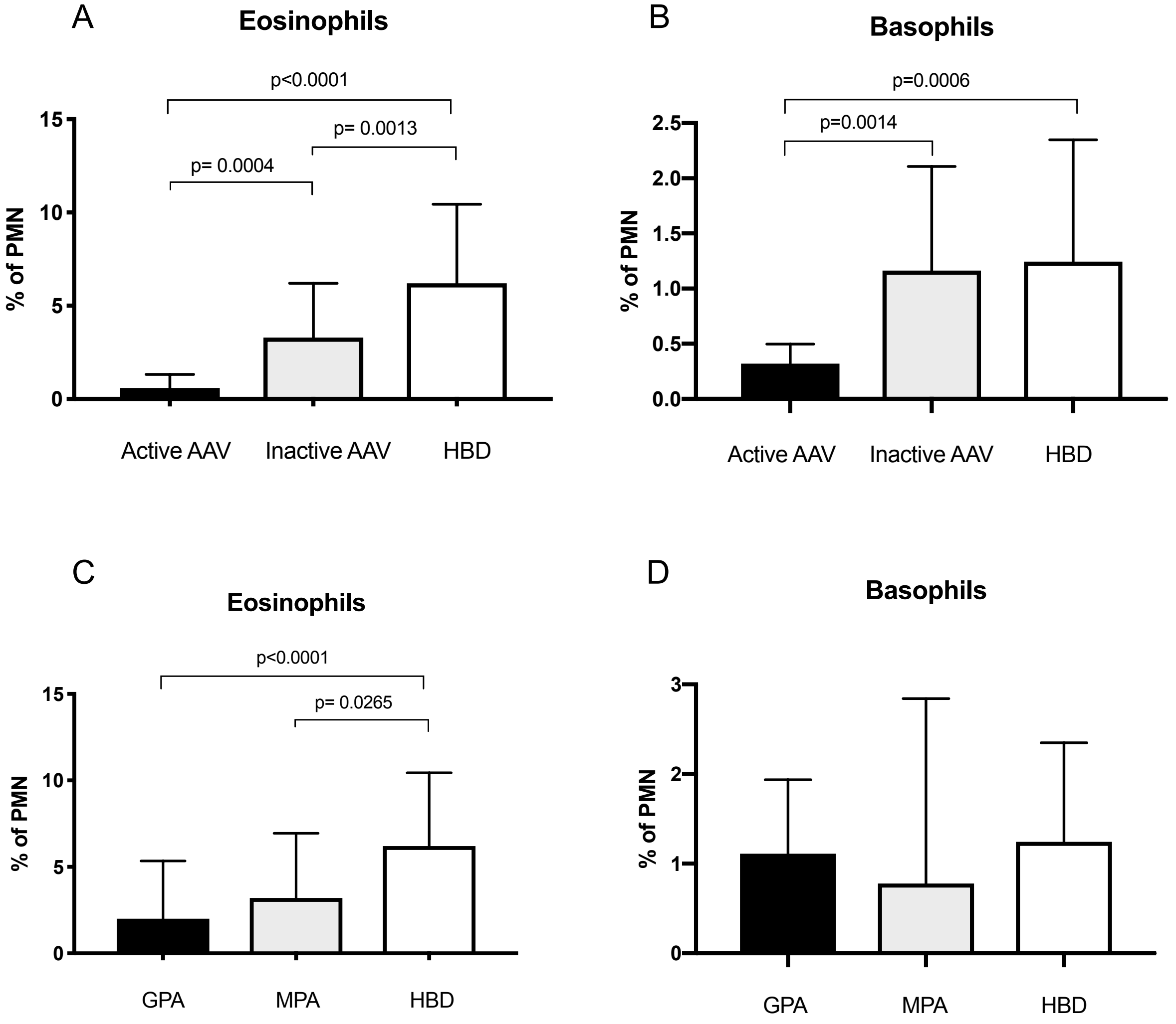

Additional file 3. The percentage of eosinophils of polymorphonuclear leukocytes (PMN) and basophils of the leukocytes populations is shown when the patients are divided into active and inactive (A and B) or into GPA or MPA (C and D). Patients with active disease had lower levels of both eosinophils and basophils but no difference were seen comparing GPA and MPA. Kruskal-Wallis test and Dunn’s multiple comparisons test was used to calculate the level of significance between the three groups. Values are reported as median ± IQR.
