# Supplementary material for: Eosinophils in anti-neutrophil cytoplasmic antibody associated vasculitis
Source: BMC Rheumatol. 2019 Mar 8;3:9. doi: 10.1186/s41927-019-0059-6 (PMC6408823; doi:10.1186/s41927-019-0059-6)
Supplement: Supplementary file 4 — The level of surface expression on eosinophils of A CD16, B CD64, C CD35, D CD193, E CD62L, F CD88, G Siglec-8, H CD11b and I CD11c was measured in healthy blood donors (HBD) and compared to anti-neutrophil cytoplasmic antibodies associated vasculitides (AAV) patients, divided into GPA and MPA patients, using flow cytometry and reported as geometric mean fluorescence intensity (MFI). Kruskal-Wallis test and Dunn’s multiple comparisons test was used to calculate the level of significance between the three groups. Values are reported as median ± IQR. No difference was seen between the GPA and MPA groups. (PDF 95 kb) [file 41927_2019_59_MOESM4_ESM.pdf]

Additional file 4

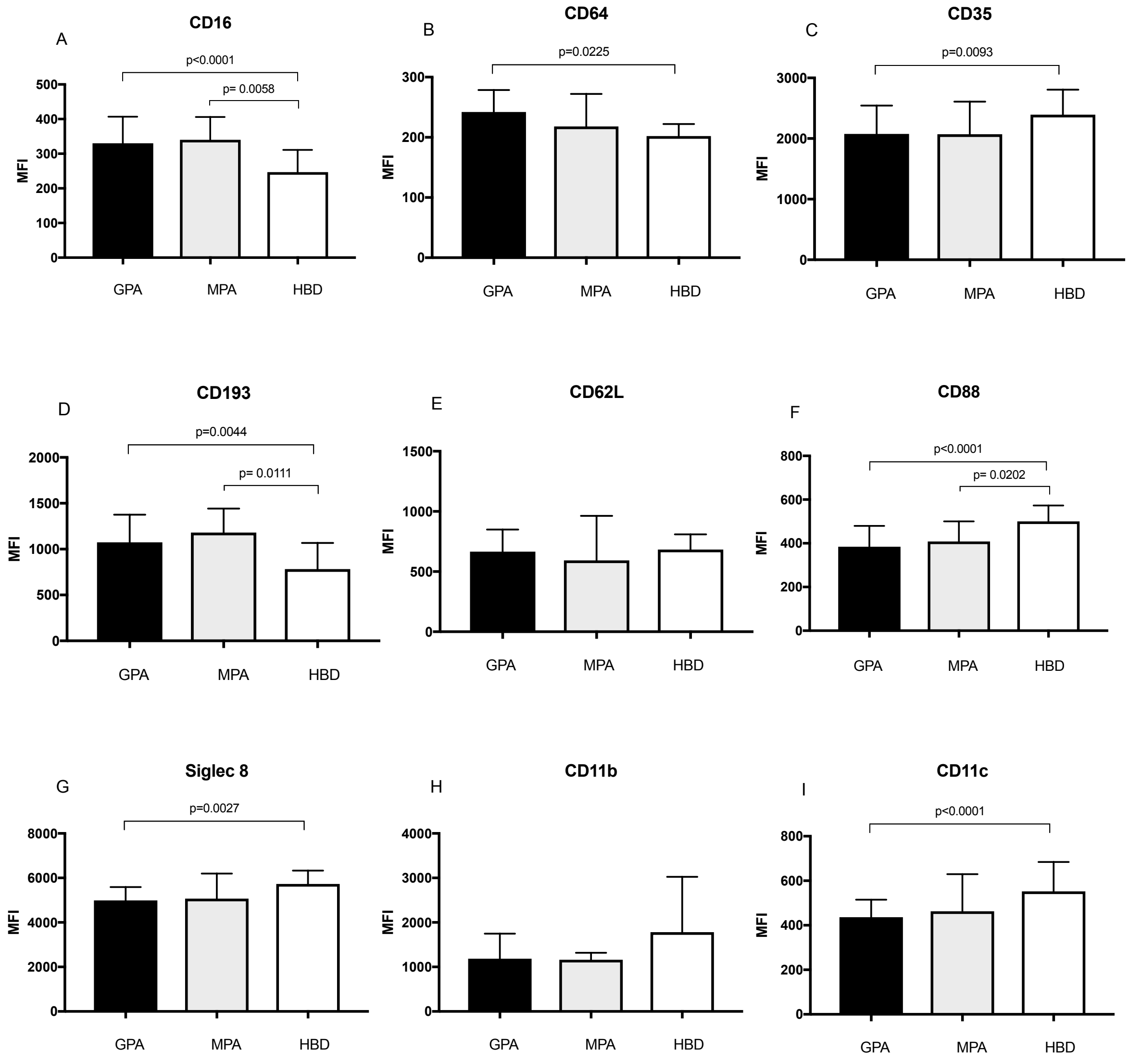

Additional file 4. The level of surface expression on eosinophils of A CD16, B CD64, C CD35, D CD193, E CD62L, F CD88, G Siglec-8, H CD11b and I CD11c was measured in healthy blood donors (HBD) and compared to anti-neutrophil cytoplasmic antibodies associated vasculitides (AAV) patients, divided into GPA and MPA patients, using flow cytometry and reported as geometric mean fluorescence intensity (MFI). Kruskal-Wallis test and Dunn's multiple comparisons test was used to calculate the level of significance between the three groups. Values are reported as median ± IQR. No difference was seen between the GPA and MPA groups.
