# Supplementary material for: Eosinophils in anti-neutrophil cytoplasmic antibody associated vasculitis
Source: BMC Rheumatol. 2019 Mar 8;3:9. doi: 10.1186/s41927-019-0059-6 (PMC6408823; doi:10.1186/s41927-019-0059-6)
Supplement: Supplementary file 5 — Light microscopy picture of two eosinophils that has formed EETs after incubation with PMA for 3 h at 37 °C and 5%CO2. The white arrow indicates the web formed by the DNA and the black arrows indicate the intact granules that remains around the plasma membrane remnants. (PDF 263 kb) [file 41927_2019_59_MOESM5_ESM.pdf]

Additional file 5

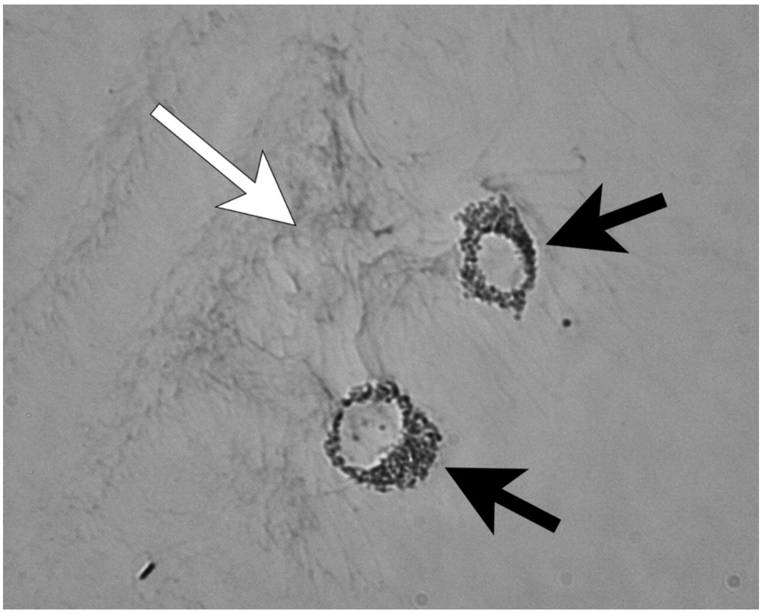

Additional file 5. Light microscopy picture of two eosinophils that has formed EETs after incubation with PMA for 3h at 37°C and 5%CO_2. The white arrow indicates the web formed by the DNA and the black arrows indicate the intact granules that remains around the plasma membrane remnants.
